# Ocrelizumab reduces cortical and deep grey matter loss compared to the S1P-receptor modulator in multiple sclerosis

**DOI:** 10.1007/s00415-023-12179-y

**Published:** 2024-01-30

**Authors:** Albulena Bajrami, Agnese Tamanti, Angela Peloso, Stefano Ziccardi, Maddalena Guandalini, Milena Calderone, Marco Castellaro, Francesca B. Pizzini, Stefania Montemezzi, Damiano Marastoni, Massimiliano Calabrese

**Affiliations:** 1 Neurology B, Department of Neurosciences, Biomedicine and Movement Sciences, University of Verona, Policlinico “G.B. Rossi” Borgo Roma Piazzale L.A. Scuro, 10, 37134 Verona, Italy; 2 Neurology Unit, Ospedale S. Chiara, Azienda Provinciale per i Servizi Sanitari (APSS), Largo Medaglie d’oro, 9, 38122 Trento, Italy; 3 Radiology Unit, Cmsr Veneto Medica S.R.L., Altavilla Vicentina, via Vicenza, 204, 36077 Vicenza, Italy; 4 Department of Diagnostics and Public Health, University of Verona, Policlinico “G.B. Rossi” Borgo Roma Piazzale L.A. Scuro, 10, 37134 Verona, Italy; 5 Department of Information Engineering, University of Padova, Via Giovanni Gradenigo, 6b , 35131 Padua, Italy

**Keywords:** Multiple sclerosis, Ocrelizumab, Brain atrophy, Cortical lesions, Neurodegeneration

## Abstract

**Introduction:**

Ocrelizumab (OCR) and Fingolimod (FGL) are two high-efficacy treatments in multiple sclerosis which, besides their strong anti-inflammatory activity, may limit neurodegeneration.

**Aim:**

To compare the effect of OCR and FGL on clinical and MRI endpoints.

**Methods:**

95 relapsing–remitting patients (57 OCR, 38 FGL) clinically followed for 36 months underwent a 3-Tesla MRI at baseline and after 24 months. The annualized relapse rate, EDSS, new cortical/white matter lesions and regional cortical and deep grey matter volume loss were evaluated.

**Results:**

OCR reduced the relapse rate from 0.48 to 0.04, FGL from 0.32 to 0.05 (both *p* < 0.001). Compared to FGL, OCR-group experienced fewer new white matter lesions (12% vs 32%, *p* = 0.005), no differences in new cortical lesions, lower deep grey matter volume loss (− 0.12% vs − 0.66%; *p* = 0.002, Cohen’s *d* = 0.54), lower global cortical thickness change (− 0.45% vs − 0.70%; *p* = 0.036; *d* = 0.42) and reduced cortical thinning/volume loss in several regions of interests, including those of parietal gyrus (*d*-range = 0.65–0.71), frontal gyrus (*d*-range = 0.47–0.60), cingulate (*d*-range = 0.41–0.72), insula (*d* = 0.36), cerebellum (cortex *d* = 0.72, white matter *d* = 0.44), putamen (*d* = 0.35) and thalamus (*d* = 0.31). The effect on some regional thickness changes was confirmed in patients without focal lesions.

**Conclusions:**

When compared with FGL, patients receiving OCR showed greater suppression of focal MRI lesions accumulation and lower cortical and deep grey matter volume loss.

**Supplementary Information:**

The online version contains supplementary material available at 10.1007/s00415-023-12179-y.

## Introduction

In multiple sclerosis (MS), it is now clear that the reduction of overt inflammatory disease activity (i.e. clinical relapses, new focal magnetic resonance imaging [MRI] lesions) should be combined with the prevention of chronic inflammation and neurodegenerative phenomena that are likely to represent the main contributors to disease progression [1]. Demonstrating the strong effects of ocrelizumab (OCR), a humanized monoclonal antibody that selectively depletes CD20+ B cells, has provided a new therapeutic avenue for relapsing–remitting (RR)MS patients [2, 3]. In this view, emerging findings suggest that besides the strong anti-inflammatory activity, OCR may contribute to limiting disability progression and cognitive impairment, slowing down neurodegeneration.

Brain atrophy is a surrogate marker of neurodegeneration, and therefore, it has been incorporated as an endpoint in several recent clinical trials in MS [4]. By evaluating the data from randomized control trials of RRMS patients, significant differences in the percentage brain volume change from week 24 to week 96 between the OCR- and the IFN β-1a-group were observed in the OPERA I, but not in the OPERA II [3]. Moreover, the recent studies have evaluated the effects of OCR in specific brain structures, showing significantly lower thalamic atrophy in RRMS patients treated with OCR as compared to IFN β-1a [5], with global and regional brain volume loss rates approaching that of healthy controls [6]. Being atrophy an MRI approach measuring tissue loss, an estimation of neurodegeneration, these findings suggested that OCR promotes the reduction of both inflammation and the progression of neurodegeneration.

However, till now, no head-to-head comparisons between OCR and another high-efficacy treatment have been conducted for regional atrophy measures. The recent observational studies showed that fingolimod (FGL), a sphingosine1-phosphate (S1P) receptor modulator, significantly reduced cortical lesion formation and GM atrophy progression compared to placebo [7, 8]. Moreover, the suggested neuroprotective effect of the S1P-receptor modulators[9] has been further supported by their recent approval for the treatment of secondary progressive MS patients [10].

In the present study, we performed a head-to-head comparison between OCR and FGL on clinical outcomes after a 3-year follow-up (FU) and MRI measures of inflammation (new focal lesions) and neurodegeneration (global/regional atrophy) over a 2-year FU.

## Methods

### Subjects

**Fig. 1:**
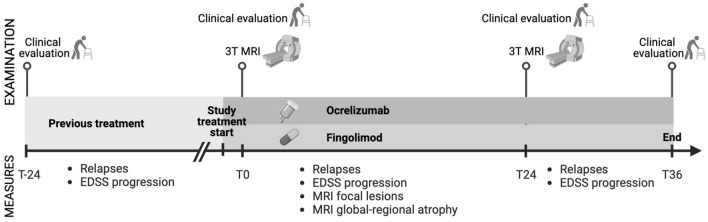
Study design. Patients underwent clinical evaluations recording relapses and EDSS during the 2 years before starting ocrelizumab or fingolimod. After starting therapy patients underwent the rebaseline 3 T-MRI (T0, after 4 months after starting the drug) and follow-up MRI after 24 months (T24) from T0. Clinical follow-up was performed till the second MRI and extended forward for 1 year to record any further relapse and to confirm the EDSS value at T24. Image created in https://www.biorender.com

In this observational, prospective, longitudinal, 3-year study, we included 95 RRMS patients: 57 treated with ocrelizumab and 38 with fingolimod (see Fig. 1 for the study design).

Inclusion criteria were: a diagnosis of relapsing–remitting MS according to McDonald criteria [11]; neurological examination every 6 months until the third year of follow-up (T36); a 3 T MRI performed at T0 (the re-baseline MRI) and after 24 months from re-baseline T24; treatment with FGL or OCR. Exclusion criteria were any condition that prevented the execution of MRI or the administration of OCR and FGL. Most patients under treatment with FGL/OCR were not-responders to previous treatment and highly active patients (28 patients), or in natalizumab at high risk of multifocal leukoencephalopathy (10 patients) or naive highly active (19 patients). Patients starting treatment with OCR were excluded if they had previously received FGL at any time point.

### Clinical evaluation

Each patient was clinically assessed by recording new relapses and the EDSS[12] at least every 6 months for an extended FU of 3 years (T0–T24–T36) and also data in the previous 24 months before starting the therapy were collected (T-24).

The annualized relapse rate (ARR) was calculated as the total number of relapses divided by the total number of years of FU.

*Disability progression* was assessed by EDSS change between T0 and T24 (Δ-EDSS) and confirmed after 6 months. *Confirmed disability progression* was defined as an EDSS increase at T24 of ≥ 1.0 in patients with EDSS at T0 ≤ 5.5 or an increase of ≥ 0.5 when the EDSS at T0 was > 5.5, sustained for at least 6 months after T24. Progression was defined as independent of relapse activity (PIRA) if there was a confirmed disability accumulation in the EDSS scale during 6 months free of relapses, whereas, it was defined as relapse-associated worsening (RAW) if occurred due to incomplete recovery after 6 months following a relapse [2]. *Confirmed disability improvement* was defined as a decrease of ≥ 1.0 points in the EDSS scale in patients with EDSS ≥ 2 and ≤ 5.5 or of ≥ 0.5 when the EDSS score was > 5.5, sustained for at least 6 months. *Stable patients* were identified as those not included in the previous categories.

*No-evidence of disease activity (NEDA)* was also evaluated. In this study, NEDA was defined as a composite score obtained from three related measures of disease activity (NEDA-3): (i) no evidence of relapses; (ii) no confirmed disability progression defined previously; and (iii) no new or enlarging T2 lesions [13, 14]. In addition, since in the present study, we also looked at the number of CLs, we included the absence of any new CLs in the definition of NEDA-3 patients.

The local ethics committee approved the present study, and informed consent was obtained from all patients.

### Image acquisition protocol at 3 T MRI

MRI sequences have been acquired by Philips Achieva 3 T MR Scanner (Philips Medical Systems, Best, The Netherlands). No software updating was carried out during the study period. The following images were acquired from each subject at T0 corresponding to re-baseline MRI (after 4 months from treatment start) and after 24 months from the re-baseline MRI, T24: (1) 3D T1 weighted sequence (MP-RAGE) TR/TE = 8000/380 ms, TI = 2360 ms, flip angle = 8°, voxel dimension = 1 × 1 × 1 mm^3^, field of view (FOV) = 240 × 240 × 180 mm^3^; (2) 3D fluid attenuated inversion recovery (FLAIR) TR/TE = 8000/288 ms, TI = 2356 ms, voxel dimension = 1 × 1 × 1 mm^3^; (3) 3D double inversion recovery (DIR) TR/TE = 5500/275 ms, TI1/TI2 = 450/2550 ms voxel dimension = 1 × 1 × 1 mm^3^.

### MRI analysis

#### Focal lesions estimation

During the study, each MRI was evaluated by the neuroradiologist for MRI reports and by two neurologists, A.B. and M.C., both well-trained and experienced in MS, blinded to patients' information. The number of pre-existing cortical lesions (CLs) and white-matter lesions (WMLs) and the number of new CLs and WMLs at T24, were assessed, respectively, on DIR and FLAIR images, following the recommendations for CL scoring in patients with MS [15]. Lesion numbers were described in classes for both types: 0–3 lesions; 4–10 lesions; 10–20 lesions, more than 20 lesions [16]. WMLs were also segmented with the lesion prediction algorithm (LPA, SPM12) and filled on T1 with the Lesion Segmentation Tool (LST) [17, 18]. Total T2-lesion load (T2-LL) was determined on FLAIR for both cohorts. The presence/absence of spinal cord lesions was recorded using clinical and MRI reports given by the neuroradiologist.

#### Regional cortical thickness/volume evaluation

Regional cortical thickness and regional volume of deep GM nuclei at T0 and after 2 years were calculated using the longitudinal stream included in the *Freesurfer* image analysis suite (release v7.1.1), available online (http://surfer.nmr.mgh.harvard.edu/) on T1-weighted lesions filled. Freesurfer QA tools were performed during each “recon” step. The weighted mean of the left and the right hemisphere, for each ROI of the *Freesurfer* parcellation (based on the Desikan–Killiany atlas), were considered for the analysis.

#### Grey matter volume change evaluation

The *Freesurfer* longitudinal pipeline also provides the rate of total GM volume. Once aligned with surface-based registration methods, T0 and T24 GM segmentation masks are used to calculate the rate of GM volume change as follows: GM volume (T24) − GM volume (T0)/GM volume (T0).

### Statistical analysis

Statistical analyses of the demographic, clinical, and global MRI variables were performed in SPSS version 28 (Chicago, IL). All variables were checked for normality with the Kolmogorov–Smirnov test and histogram inspection. Variables were reported as mean and standard deviation (SD), median and interquartile range, or count and relative frequencies, accordingly.

Multivariate general linear model (GLM) analyses were performed to assess group differences (OCR vs FGL) in clinical and MRI variables, with sex and age entered as covariates. Bonferroni’s corrected values of *p* < 0.05 were considered statistically significant. Non-normal distributed or categorical variables were compared between the groups using Mann–Whitney tests or Pearson Chi-Square as indicated. To assess within-group longitudinal changes, paired *t* tests or Wilcoxon Signed Ranks were used as appropriate. We computed Cohen’s *d* as the difference between the two groups’ mean divided by the adjusted standard deviation of the measurement. For the clinical outcomes of relapses and EDSS change, Kaplan–Meier curves were used with time from the first administration of OCR and FGL as timescales.

## Results

### Study population

The main baseline demographic and clinical characteristics of the patients who ended the FU are summarised in Table 1. The two groups, OCR and FGL, were well balanced for demographics and focal MRI variables; however, certain differences in baseline characteristics identified OCR as those patients with a worst prognostic disease course: significant higher proportion of male patients (32% vs 16% for FGL), higher EDSS score at baseline (median = 4.0 vs 2.25 for FGL), higher annualised relapse rate before starting the therapy (mean = 0.48 vs 0.32 for FGL) and higher number of spinal cord lesions (mean = 1.42 vs 0.4 for FGL).

**Table 1:** Demographics and disease characteristics at baseline and 24 months before starting the drugs

Demographics and disease characteristics	OCR (*N* = 57)	FGL^b^ (*N* = 38)
Male sex, no. (%)	16 (32%)	6 (16%)*
Age at onset, yr	30.2 ± 9.90	29.0 ± 9.1
Age at first MRI, yr	41.6 ± 9.9	39.5 ± 9.6
MS type at onset RR/SP/PP, *N*	57/0/00	38/0/0
Time since symptom onset, yr	10.8 ± 8.1	9.2 ± 6.7
Time since diagnosis, yr	8.5 ± 7.5	6.3 ± 5.4
Presence of OCB, *N* (%)	37 (65%)	24 (63.2%)
Disability 24 mo. before therapy and at T0		
EDSS 24 mo. before therapy^a^	3.0 [2.0–4.7]	2.0 [1.5–3.0]*
EDSS change 24 mo. before therapy^a^	0.5[0.0–1.0]	0.0 [0.0–0.0]
EDSS T0^a^	4.0 [3.0–5.7]	2.25 [1.5–3.0]***
Disability progression	24 (42%)	4 (42%)**
Disease activity 24 mo. before therapy		
Patients with relapses, *N* (%)	51 (74%)	22 (58%)
Number of relapses	0.96 ± 0.60 (0–2)	0.63 ± 0.59 (0–2)*
ARR	0.48 ± 0.30	0.32 ± 0.29*
MRI characteristics at T0		
Number of spinal lesions	1.4 ± 1.8 (0–7)	0.4 ± 0.7 (0–3)**
Number of WMLs (%)		
Classes < 4/4–10/10–20/ > 20	0/10/30/60	0/8/11/81
Number of CLs (%)		
Classes < 4/4–10/10–20/ > 20	11/30/17/42	13/32/21/34

Comparison in-between groups have been performed using *t* tests, Mann–Whitney test, chi-square or GLM with sex, age and disease duration as covariates and Bonferroni correction

*yr* years, *no* number, *mo* months, *RR* relapsing–remitting, *SP* secondary progressive, *PP* primary progressive, *OCB* oligoclonal bands, *EDSS* expanded disability status scale, *ARR* annualized relapse-rate, *WM* white matter, CL cortical lesions

Data are reported as mean ± standard deviation except where otherwise reported

Significance is reported as following *p < 0.05; **p < 0.01; **p < 0.001

^a^(Median [IQR])

^b^Significance is reported relative to OCR group

### Effectiveness of OCR when compared with FGL

For clinical and focal MRI endpoints at the end of the study, see Table 2.

**Table 2:** Clinical and MRI focal lesions end points at the end of follow-up

Clinical and MRI endpoints	OCR (*N* = 57)	FGL^b^ (*N* = 38)
Relapses at T24 and T36		
Patients with relapses, *N* (%)	6 (11%)	6 (16%)
ARR at T36	0.04 ± 0.10	0.05 ± 0.12
ARR during T0–T24	0.05 ± 0.16	0.00 ± 0.00
ARR during T24–T36	0.00 ± 0.00	0.16 ± 0.37***
Clinical disability at T24		
EDSS T24^a^	4.5 [2.0–6.0]	2.5 [1.4–3.0]***
EDSS change 24 mo. after therapy^a^	0.0[0.0–0.5]	0.0[0.0–0.0]
Clinically stable at T24		
Patients with event, *N* (%)	35 (62%)	30 (79%)
Disability progression at T24		
Patients with event, *N* (%)	15 (26%)	4 (10.5%)
Disability improvement at T24		
Patients with event, *N* (%)	7 (12%)	4 (10.5%)
MRI characteristics at T24		
New WMLs T24 yes, *N* (%)	7 (12%)	12 (32%)*
New CLs T24 yes/no, *N* (%)	10 (18%)	8 (21.1%)
NEDA at T24 and T36		
NEDA3/EDA3 T24 (%)	58/42%	63/37%
NEDA3/EDA3 T36 (%)	58/42%	58/42%

Data are reported as mean ± standard deviation if not differently reported

Comparison in-between groups have been performed using *t* tests, Mann–Whitney test, chi-square or GLM with sex, age and disease duration as covariates and Bonferroni correction

*yr* years, *no* number, mo months, *RR* relapsing–remitting, *SP* secondary progressive, *PP* primary progressive, *OCB* oligoclonal bands, *EDSS* expanded disability status scale, *ARR* annualized relapse-rate, *WM* white matter, *CL* cortical lesions, *NEDA* no evidence of disease activity, *EDA* evidence of disease activity

Significance is reported as following *p < 0.05; **p < 0.01; **p < 0.001

^a^Data are reported as median [IQR]

^b^Significance is reported relative to OCR group

#### Annualized relapse rate

**Fig. 2:**
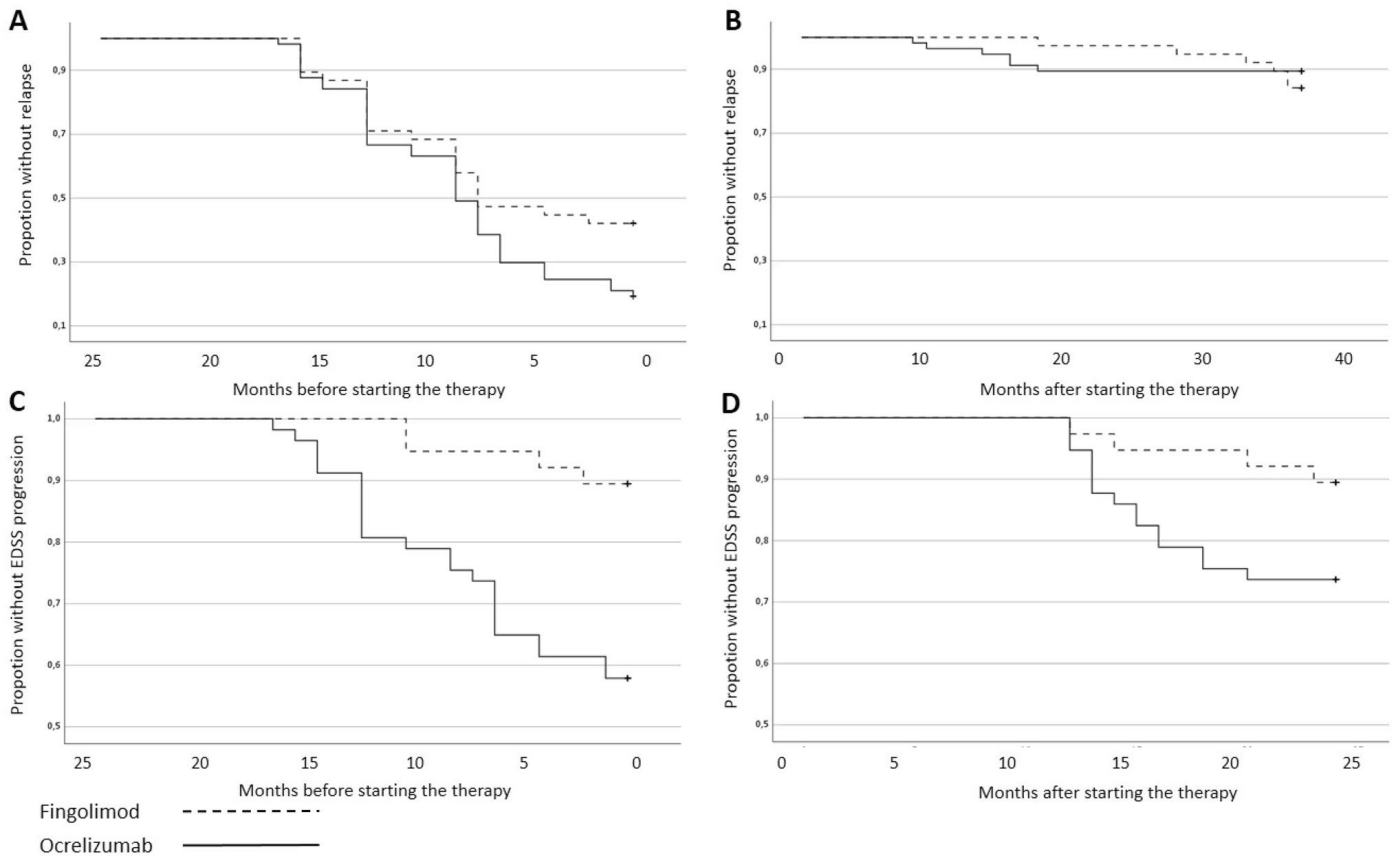
Plots depicting the relapses and EDSS outcomes for the two cohorts Kaplan–Meier curve for the fingolimod and ocrelizumab groups **A** for time until clinical relapses the previous 2 years before starting treatment, **B** for time until clinical relapses within the first 3 years after treatment. **C** EDSS progression in the 2 years before starting the treatment and **D** after starting the drug

A total of 89% patients with OCR and 84% with FGL were free of relapses at 3-year FU, not significantly different in-between the two cohorts. The decrease over time in proportion of patients without clinical relapse before and after the therapy is shown respectively in Fig. 2A, B. Both treatments significantly reduced the ARR: at T36, mean ARR reduced from 0.48 to 0.04 in OCR (*p* < 0.001, *d* = 1.4), and from 0.32 to 0.05 in FGL-group (*p* < 0.001, *d* = 0.91). In OCR all the relapses occurred in the first months of therapy (the median time before the first relapse was 14 [range 8.8–15.5] months), whereas in the FGL-group only 3% patients had relapses before T24 and the remanent 13% occurred after the 2 years of therapy (the median before the first relapse was 33 [range 24.5–36] months).

#### Disability

At 2-year FU, 42 patients (74%) in OCR and 34 patients (89.5%) in FGL were free of disability worsening and the two patient groups did not show significant differences.

The OCR patients experienced significantly higher EDSS change before starting the therapy as compared to the 2 years after (median EDSS change 0.5 vs 0.0, *p* = 0.002, respectively before and after 2 years of FU): OCR at T0 experienced a significantly increased EDSS score as compared to 2 years before [T-24] (3.0 vs 4.0, *p* < 0.001), whereas the EDSS did not change substantially between T0 and after 2 years of follow-up [T24] (4.0 vs 4.5, *p* = 0.21).

At 2-year FU, 15 patients (26%) in OCR and 4 patients (10.5%) in FGL experienced disability worsening. In OCR-group 12 out of 15 (80% of patients with progression), whereas in FGL-group 2 out of 4 (50% of patients with progression) experienced progression independent of relapse activity at 2-year FU (*p* value in between groups not significant *p* = 0.10). Therefore only 3 out of 15 in OCR and 2 out of 4 in FGL experienced RAW (not significant in-between groups). The decrease over time of the proportion of patients without EDSS progression before and after starting the therapy is shown respectively in Fig. 2C, D.

#### MRI focal lesions

At 2-year FU, 88% of OCR patients, 68% of FGL patients (*p* = 0.005) were free of MRI evidence of new or enlarging hyperintense lesions on T2-weighted images. In contrast, no differences in the percentage of patients free of new CLs was reported (82% vs. 79%, *p* = 0.64).

#### No-evidence of disease activity 3 (NEDA3)

No statistically significant differences in EDA/NEDA at T36 in-between groups were seen, with most patients remaining NEDA (58% in OCR, 58% in FGL).

### Grey matter atrophy and regional cortical thickness

**Fig. 3:**
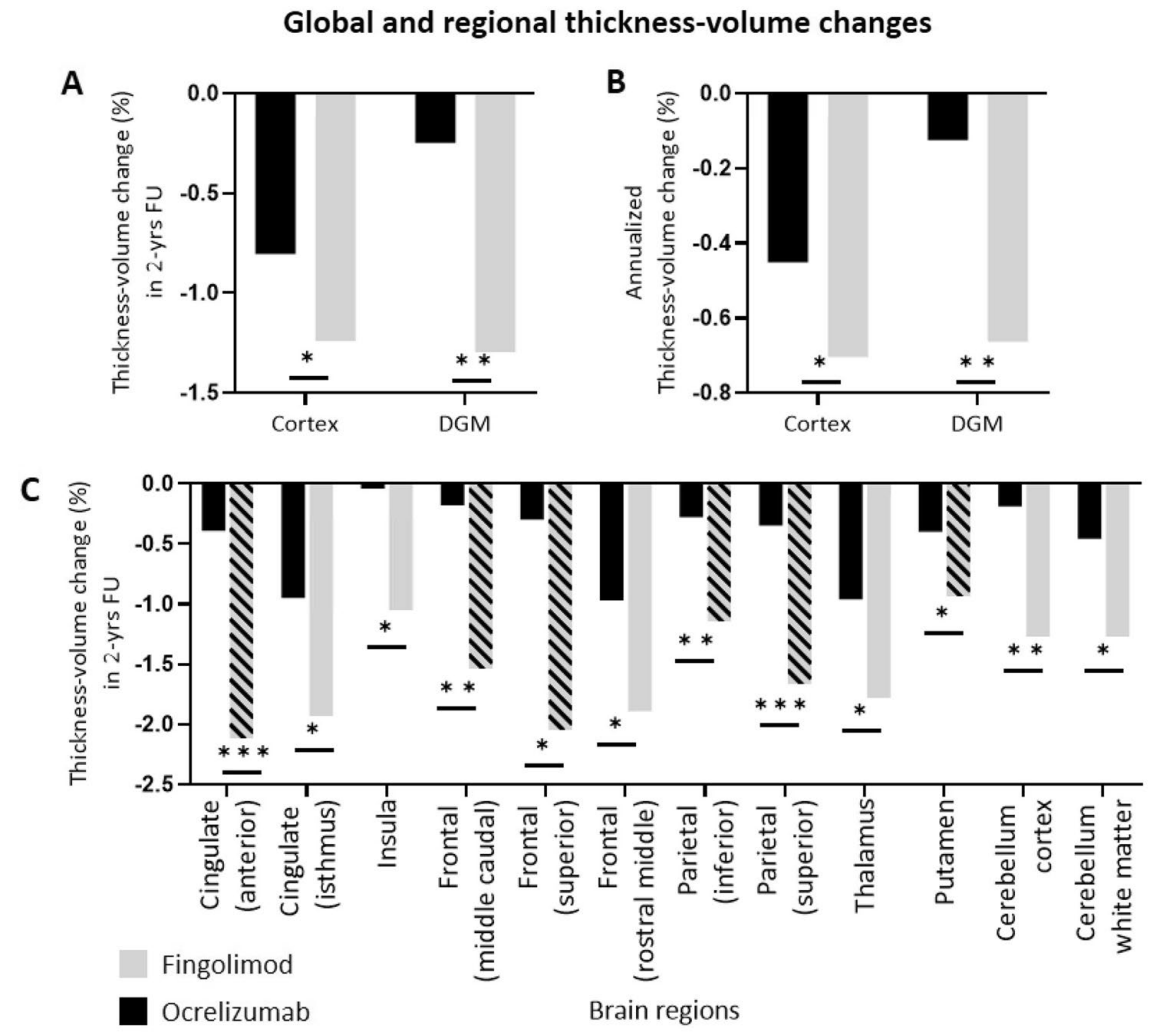
Global and regional thickness-volume changes. Mean cortical thickness and deep grey matter volume changes are depicted in (**A**), whereas the mean annualized changes are depicted in (**B**). In **C** the mean regional volume-thickness changes. The bars filled with line patterns refer to the brain regions which result significantly different also in the groups without new WM lesions during the follow-up. Significance is reported as following *p < 0.05; **p < 0.01; **p < 0.001

Global/regional atrophy data at T24-T0 are reported in Table 3 and Fig. 3. The global and regional cortical thickness/volume at T0 in both groups are reported in Supplementary Table 1.

**Table 3:** Regional/global cortical thickness change and deep grey matter volume loss, T24–T0

Brain regions	OCR (*N* = 57)	FTY (*N* = 38)		*p* value	Cohen’s *d*
Global volume/thickness change (%)					
	Cortical thickness	− 0.80 ± 0.96	− 1.24 ± 0.98	0.019	0.47
	Cortical thickness (annualized)	− 0.45 ± 0.61	− 0.70 ± 0.60	0.036	0.42
	Deep grey matter volume	− 0.25 ± 1.41	− 1.30 ± 1.66	< 0.001	0.71
	Deep grey matter volume (annualized)	− 0.12 ± 0.80	− 0.66 ± 0.89	0.002	0.54
Regional cortical thickness changes (%)					
	Superior parietal	− 0.35 ± 2.57	− 1.78 ± 1.96	< 0.001	0.71
	Inferior parietal	− 0.32 ± 1.91	− 1.47 ± 1.79	0.003	0.65
	Caudal middle frontal	− 0.51 ± 2.64	− 1.91 ± 2.66	0.004	0.60
	Superior frontal	− 0.23 ± 2.36	− 1.40 ± 1.83	0.010	0.54
	Rostral middle frontal	− 0.70 ± 2.70	− 1.95 ± 2.12	0.034	0.47
	Caudal anterior cingulate	− 0.23 ± 3.01	− 1.84 ± 1.93	< 0.001	0.72
	Isthmus cingulate	− 0.78 ± 2.63	− 1.61 ± 2.10	0.046	0.41
	Insula	− 0.40 ± 2.00	− 0.94 ± 1.63	0.021	0.36
Regional deep-grey matter volume changes (%)					
	Cerebellar cortex	− 0.20 ± 2.40	− 1.78 ± 3.34	0.005	0.72
	Cerebellum white matter	− 0.44 ± 3.16	− 1.65 ± 4.08	0.014	0.44
	Thalamus	− 0.99 ± 2.44	− 1.78 ± 2.70	0.032	0.31
	Putamen	− 0.41 ± 1.85	− 1.24 ± 2.45	0.043	0.35

Data are reported as mean ± standard deviation of % changes; only significant changes are reported in the table

Comparison in-between groups have been performed GLM with sex, age and disease duration as covariates and Bonferroni’s correction. The effect size is reported as Cohen’s *d* obtained as the difference of the means divided by the standard deviation of the data

#### Global volume changes

At T24 comparing RRMS patients treated with OCR and FGL the first group showed significantly less annualized deep grey matter volume loss (mean change − 0.12 vs − 0.66, *p* = 0.002, *d* = 0.54) annualized cortical thickness change (mean change − 0.45 vs − 0.70, *p* = 0.036, *d* = 0.42).

#### Regional volume changes

Among the 37 regions studied, OCR_,_ compared to FGL showed less atrophy in all regions, among which those statistically significant were the cingulate cortex (caudal anterior *p* < 0.001, *d* = 0.72; isthmus *p* = 0.046, *d* = 0.41), the frontal gyrus (caudal middle *p* = 0.004, *d* = 0.60; superior *p* = 0.010, *d* = 0.54; rostral middle *p* = 0.034, *d* = 0.47), the inferior parietal (*p* = 0.003, *d* = 0.65) and superior parietal (*p* < 0.001, *d* = 0.71), and the insula (*p* = 0.021, *d* = 0.36). Among the deep grey matter regions, the two groups differed for volume changes in the thalamus (*p* = 0.032, *d* = 0.31), putamen (*p* = 0.043, *d* = 0.35), cerebellar cortex (*p* = 0.005, *d* = 0.72) as well as cerebellar white matter (*p* = 0.014, *d* = 0.44).

#### Volume changes in patients without new focal lesions

The same analysis was performed also in the subgroup of patients without new focal inflammatory lesions (both WM and GM), 88% of OCR patients (*N* = 50) and 68% of FGL patients (*N* = 26). When compared with FGL, the OCR subgroup confirmed less atrophy in the following regions: the caudal anterior cingulate cortex (*p* < 0.001, *d* = 0.71), the caudal middle frontal gyrus (*p* = 0.005, *d* = 0.64), the superior frontal gyrus (*p* = 0.023, *d* = 0.54), the inferior parietal gyrus (*p* = 0.047, *d* = 0.49) and superior parietal gyrus (*p* = 0.004, *d* = 0.71). In the deep grey matter only the putamen atrophy was significantly different (*p* = 0.038, *d* = 0.33) (see in Fig. 3C, bars with line pattern).

The new lesion accumulation correlated with the volume loss in cerebellum cortex (*r* = − 0.34, *p* < 0.001), thalamus (*r* = − 0.34, *p* < 0.001), nucleus caudate (*r* = − 0.54, *p* < 0.001) and the hippocampus (*r* = − 0.37, *p* < 0.001). No significant correlations were seen with cortical thinning or global volume loss.

## Discussion

This is a single-centre effectiveness comparative study between ocrelizumab and another high-efficacy treatment (fingolimod) in a real-world setting for an FU of 3 years. We investigated common clinical variables but also conventional and non-conventional MRI outcomes: focal inflammatory WM lesions, cortical lesions, global and regional brain volume and cortical thickness changes. Our cohort consisted of a heterogeneous group of patients with a large variety in age and disease activity.

The annualized relapse rate decreased in OCR and FGL, in line with the relapse rate at the 2-year FU of the respective drug trials [3, 19]. However, we did not report significant differences between the two treatment groups for clinical relapses, disability worsening, and percentage of patients reaching NEDA-3 at the end of follow-up. These results slightly differed from those in the previous studies, showing the superiority of anti-CD20 drugs in preventing relapses [20, 21]. However, this might be due partly to the prognostically worse baseline characteristics of OCR patients as compared to FGL: higher EDSS score (median EDSS at baseline OCR 4.0 vs FGL 2.25), a higher proportion of male patients, but also a greater involvement of the spinal cord. Interestingly, in the OCR group, the relapses occurred early during the FU, whereas in the fingolimod, they occurred later in the observational period. This difference might be due to a faster lymphocyte redistribution in patients with fingolimod than those with ocrelizumab. Therefore, the OCR group might have a higher risk of early relapse, especially if patients at baseline showed high disease disability. Indeed, in a recent study, the authors showed that patients with OCR with baseline EDSS ≥ 4 (the case of our OCR cohort) had a higher risk of disability worsening and lower NEDA-3 rates at 2-year FU [22].

Although, in line with the literature, OCR showed a greater reduction of overt inflammatory disease activity in terms of MRI focal WM lesions (at 2-year FU, 88% OCR vs 68% FGL) [20, 21] but not a statistically different accrual of new CLs. It is known that patients have very few new visible CLs in 2 years of FU and under high-efficacy treatment and, therefore, not enough for a meaningful statistical difference.

Nowadays, it is clear that, in estimating the drug efficacy, we should assess, besides the reduction of inflammatory activity and clinical outcomes, the prevention of neurodegenerative phenomena, which are difficult to evaluate clinically, but can be estimated in vivo by measures of global and regional atrophy on MRI. Our study showed a superiority of ocrelizumab to fingolimod in slowing down brain atrophy globally and in specific key regions.

The brain regions which seem to be more influenced by the medication were those which are also, the most affected by the disease: in the cortex, the parietal gyrus, the frontal gyrus, the cingulate cortex and the insula, whereas in the deep grey matter, mainly cerebellum, putamen and thalamus. Pathology studies demonstrated that these regions are the predominant sites of grey matter and, to a lesser extent, of white matter demyelination and the brain areas with the highest presence of cortical lesions. In particular, among all the regions, the cingulate gyrus seemed to have the most prominent grey and white matter demyelination [23], which might lead to the atrophy seen by MRI.

Neurodegeneration in MS might be initially triggered by inflammation and afterwards self-maintained by the persistent compartmentalised inflammation, especially if there is limited drug access to the neuronal compartment after the blood–brain barrier integrity is restored. Moreover, different studies showed the key role of B-cell immunity in the biological mechanisms underlying cortical pathology [24]. Therefore, ocrelizumab, targeting the CD20 marker on B lymphocytes and restraining the immune cell circulation from blood to the CNS, might play a crucial role in limiting the establishment of intrathecal inflammation and, consequently, neurodegeneration processes. This effect might have its best expression when the drug is administered from the earliest phases of the disease.

Moreover, the cingulate cortex and insula have, also, extensive connections with other regions and the putamen receives significant inputs from the motor cortex. Therefore, possible additional factors for their early atrophy can include disconnection secondary to white matter lesions. Consequently, ocrelizumab, suppressing the accrual of new WM lesions, might limit retrograde neurodegeneration.

In addition, the inflammatory demyelination may also result from activation of innate inflammatory cells (particularly microglia) within established focal WM lesions and normal-appearing tissues. These lesions, termed “chronic active,” also reflect a compartmentalized chronic inflammation that has been suggested to contribute to MS severity and progression. The accumulation of chronic active lesions may represent one of the contributors to disability independent of relapses (i.e. PIRA). Ocrelizumab reducing the chronic activity in pre-existing lesions [25] might also limit the ongoing demyelination in normal-appearing tissues; however, until now; this role has been shown to be the modest; this could explain why in our study the effect of the OCR on disability progression was less clear than that on the disease activity and why the disability progression in the majority of patients was relapse-independent.

Recently, the crucial role of the CSF cytokines in MS and its relationship with cortical pathology is also emerging [26]. A strong association has been observed between high levels of CSF chemokines related to lymphoid neogenesis and B-cells with cortical damage accumulation over 4 years [27]. This potential role might be fundamental especially in those regions, such as the thalamus and cerebellum, in anatomical proximity to CSF and whose atrophy has been correlated with CSF inflammatory profile [28]. Ocrelizumab, limiting the B-cell driven intrathecal inflammation, indirectly might reduce proinflammatory cytokines in the CSF, slowing the worsening of cortical pathology and, therefore the long-term disability accumulation.

We also looked at the atrophy differences in patients without radiological signs of disease activity (without new WMLs and CLs) to exclude the effect of lesion accumulation on brain volume change.

As expected, we found a correlation between the lesion accumulation and the volume loss in several GM regions. This confirms that the neuroprotective effect of treatment is mainly driven by the lower anterograde/retrograde GM degeneration consequent to the focal lesions. The regions showing significant volume loss differences between subgroups are less than the comparisons considering the entire group, suggesting still an important and well-known role of the drug on the acute inflammatory component of the disease. However, despite excluding patients with acute disease activity, the OCR group still showed lower cortical and deep GM volume loss in several cortical and deep GM regions, including the anterior cingulate and the putamen. This result seems to suggest that the neuroprotective effect of OCR might be at least partly independent of the focal inflammation and that the treatment might be active also on the smoldering component of MS.

From a clinical point of view, the regions in this study particularly affected by the treatment are also those which correlate with cognitive impairment in MS [29]. Indeed, recent studies [30, 31], by evaluating pooled data from approval trials, showed the positive effects of ocrelizumab on cognitive functions. Therefore, ocrelizumab may exert beneficial effects not only by suppressing inflammatory activity in terms of reducing cognitive relapses, but also possibly limiting the neurodegenerative processes independent of overt inflammation [32].

This study has several limitations, including the relatively short follow-up and the consequent difficulty in measuring the brain volume changes; we can’t exclude the presence of “pseudoatrophy”, however, using a rebaseline MRI we limited this problem, however it might be necessary a longer follow-up to adequately evaluate this effect.

In conclusion, the limitation of persistent compartmentalised inflammation, the interference with the activation of innate inflammation in pre-existing lesions and the reduction of retrograde neurodegeneration might be the main underlying mechanisms of the efficiency of ocrelizumab on neurodegenerative processes, shown indirectly as a diminished volume loss compared to another drug.

### Supplementary Information

Below is the link to the electronic supplementary material.

Supplementary file1 (DOCX 15 KB)

## References

[1] Faissner S, Plemel JR, Gold R, Yong VW (2019) Progressive multiple sclerosis: from pathophysiology to therapeutic strategies. Nature Reviews Drug Discovery, vol 18 10.1038/s41573-019-0035-2 31399729

[2] Kappos L, Wolinsky JS, Giovannoni G, Arnold DL, Wang Q, Bernasconi C (2020). Contribution of relapse-independent progression vs relapse-associated worsening to overall confirmed disability accumulation in typical relapsing multiple sclerosis in a pooled analysis of 2 randomized clinical trials. JAMA Neurol.

[3] Hauser SL, Bar-Or A, Comi G, Giovannoni G, Hartung H-P, Hemmer B (2017). Ocrelizumab versus interferon beta-1a in relapsing multiple sclerosis. N Engl J Med.

[4] Sormani MP, Arnold DL, De Stefano N (2014). Treatment effect on brain atrophy correlates with treatment effect on disability in multiple sclerosis. Ann Neurol.

[5] Arnold DL, Sprenger T, Bar-or A, Wolinsky JS, Kappos L, Kolind S et al (2022) Ocrelizumab reduces thalamic volume loss in patients with RMS and PPMS, pp 1–10 10.1177/13524585221097561 PMC9493406 35672926

[6] Kolind S, Gaetano L, Assemlal HE, Bernasconi C, Bonati U, Elliott C, Hagenbuch N, Magon S, Arnold DL, Traboulsee A (2023). Ocrelizumab-treated patients with relapsing multiple sclerosis show volume loss rates similar to healthy aging. Mult Scler.

[7] Yousuf F, Dupuy SL, Tauhid S, Chu R, Kim G, Tummala S (2017). A two-year study using cerebral gray matter volume to assess the response to fingolimod therapy in multiple sclerosis. J Neurol Sci.

[8] Bajrami A, Pitteri M, Castellaro M, Pizzini F, Romualdi C, Montemezzi S (2018). The effect of fingolimod on focal and diffuse grey matter damage in active MS patients. J Neurol.

[9] Pitteri M, Magliozzi R, Bajrami A, Camera V, Calabrese M (2018) Potential neuroprotective effect of Fingolimod in multiple sclerosis and its association with clinical variables, vol 19, Expert Opinion on Pharmacotherapy. Taylor and Francis Ltd, pp 387–95 10.1080/14656566.2018.1434143 29397790

[10] Kappos L, Bar-Or A, Cree BAC, Fox RJ, Giovannoni G, Gold R (2018). Siponimod versus placebo in secondary progressive multiple sclerosis (EXPAND): a double-blind, randomised, phase 3 study. Lancet.

[11] Polman CH, Reingold SC, Banwell B, Clanet M, Cohen JA, Filippi M (2011). Diagnostic criteria for multiple sclerosis: 2010 revisions to the McDonald criteria. Ann Neurol.

[12] Kurtzke JF (1983) Rating neurologic impairment in multiple sclerosis: an expanded disability status scale (EDSS), pp 1444–53 10.1212/wnl.33.11.1444 6685237

[13] Havrdova E, Galetta S, Stefoski D, Comi G (2010). Freedom from disease activity in multiple sclerosis. Neurology.

[14] Giovannoni G, Turner B, Gnanapavan S, Offiah C, Schmierer K, Marta M (2015). Is it time to target no evident disease activity (NEDA) in multiple sclerosis?. Mult Scler Relat Disord.

[15] Geurts JJG, Roosendaal SD, Calabrese M, Ciccarelli O, Agosta F, Chard DT (2011). Consensus recommendations for MS cortical lesion scoring using double inversion recovery MRI. Neurology.

[16] Tintore M, Rovira À, Río J, Otero-Romero S, Arrambide G, Tur C, Comabella M, Nos C, Arévalo MJ, Negrotto L, Galán I, Vidal-Jordana A, Castilló J, Palavra F, Simon E, Mitjana R, Auger C, Sastre-Garriga J, Montalban X (2015). Defining high, medium and low impact prognostic factors for developing multiple sclerosis. Brain.

[17] Schmidt P, Gaser C, Arsic M, Buck D, Förschler A, Berthele A (2012). An automated tool for detection of FLAIR-hyperintense white-matter lesions in Multiple Sclerosis. Neuroimage.

[18] Valverde S, Oliver A, Lladó X (2014). A white matter lesion-filling approach to improve brain tissue volume measurements. NeuroImage Clin.

[19] Kappos L, Radue EW, O'Connor P, Polman C, Hohlfeld R, Calabresi P, Selmaj K, Agoropoulou C, Leyk M, Zhang-Auberson L, Burtin P, FREEDOMS Study Group (2010). A placebo-controlled trial of oral fingolimod in relapsing multiple sclerosis. N Engl J Med.

[20] Alping P, Frisell T, Novakova L, Islam-Jakobsson P, Salzer J, Björck A, Axelsson M, Malmeström C, Fink K, Lycke J, Svenningsson A, Piehl F (2016). Rituximab versus fingolimod after natalizumab in multiple sclerosis patients. Ann Neurol.

[21] Bigaut K, Kremer L, Fabacher T, Ahle G, Goudot M, Fleury M, Gaultier C, Courtois S, Collongues N, de Seze J (2022). Ocrelizumab versus fingolimod after natalizumab cessation in multiple sclerosis: an observational study. J Neurol.

[22] Cellerino M, Boffa G, Lapucci C, Tazza F, Sbragia E, Mancuso E, Bruschi N, Minguzzi S, Ivaldi F, Poirè I, Laroni A, Mancardi G, Capello E, Uccelli A, Novi G, Inglese M (2021). Predictors of ocrelizumab effectiveness in patients with multiple sclerosis. Neurotherapeutics.

[23] Bø L, Vedeler CA, Nyland HI, Trapp BD, Mørk SJ (2003). Subpial demyelination in the cerebral cortex of multiple sclerosis patients. J Neuropathol Exp Neurol.

[24] Magliozzi R, Howell O, Vora A, Serafini B, Nicholas R, Puopolo M, Reynolds R, Aloisi F (2007). Meningeal B-cell follicles in secondary progressive multiple sclerosis associate with early disease onset and severe cortical pathology. Brain.

[25] Elliott C, Belachew S, Wolinsky JS, Hauser SL, Kappos L, Barkhof F, Bernasconi C, Fecker J, Model F, Wei W, Arnold DL (2019). Chronic white matter lesion activity predicts clinical progression in primary progressive multiple sclerosis. Brain.

[26] Magliozzi R, Howell OW, Nicholas R, Cruciani C, Castellaro M, Romualdi C, Rossi S, Pitteri M, Benedetti MD, Gajofatto A, Pizzini FB, Montemezzi S, Rasia S, Capra R, Bertoldo A, Facchiano F, Monaco S, Reynolds R, Calabrese M (2018). Inflammatory intrathecal profiles and cortical damage in multiple sclerosis. Ann Neurol.

[27] Magliozzi R, Scalfari A, Pisani AI, Ziccardi S, Marastoni D, Pizzini FB, Bajrami A, Tamanti A, Guandalini M, Bonomi S, Rossi S, Mazziotti V, Castellaro M, Montemezzi S, Rasia S, Capra R, Pitteri M, Romualdi C, Reynolds R, Calabrese M (2020). The CSF profile linked to cortical damage predicts multiple sclerosis activity. Ann Neurol.

[28] Bajrami A, Magliozzi R, Pisani AI, Pizzini FB, Crescenzo F, Marastoni D, Calabrese M (2022). Volume changes of thalamus, hippocampus and cerebellum are associated with specific CSF profile in MS. Mult Scler.

[29] Calabrese M, Rinaldi F, Grossi P, Gallo P (2011). Cortical pathology and cognitive impairment in multiple sclerosis. Expert Rev Neurother.

[30] Benedict R, De Seze J, Hauser S, Kappos L, Wolinsky J, Zheng H et al (eds) (2018) Impact of ocrelizumab on cognition in patients at increased risk of progressive disease (P1.420). AAN

[31] Cohan S, Benedict R, De Seze J, Hauser S, Kappos L, Wolinsky J et al (eds) (2018) Time to cognitive worsening in patients with relapsing multiple sclerosis in ocrelizumab phase III trials (S44.005). AAN. https://n.neurology.org/content/90/15_Supplement/ S44.005 Platform Presentation S44.005. Accessed 8 Nov 2021

[32] Benedict RH, Pol J, Yasin F, Hojnacki D, Kolb C, Eckert S (2021). Recovery of cognitive function after relapse in multiple sclerosis. Mult Scler.

