# Supplementary material for: Ocrelizumab reduces cortical and deep grey matter loss compared to the S1P-receptor modulator in multiple sclerosis
Source: J Neurol. 2024 Jan 30;271(5):2149–58. doi: 10.1007/s00415-023-12179-y (PMC11055717; doi:10.1007/s00415-023-12179-y)
Supplement: Supplementary file 1 — Supplementary file1 (DOCX 15 KB) [file 415_2023_12179_MOESM1_ESM.docx]

| **Supplementary Table 1. Regional/Global cortical thickness and deep grey matter volume at baseline, T0.** | | | | | | |
| --- | --- | --- | --- | --- | --- | --- |
| **Brain regions** | | **OCR**  **(*N* = 69)** | **FGL**  **(*N* = 38)** | ***p* - Value** | **Cohen’s *d*** | |
| ***Global volume/thickness*** | |  |  |  |  | |
|  | NBV | 0.71±0.04 | 0.73±0.03 | 0.120 | / | |
|  | NGMV | 0.40±0.02 | 0.41±0.02 | 0.186 | / | |
|  | Cortical thickness (mm) | 2.38±0.09 | 2.48±0.08 | < 0.001 | 1.17 | |
|  | DGMV (x10^3^) (mm3) | 7.20±0.63 | 7.30±0.50 | 0.840 | / | |
| ***Regional thickness*** (mm) | |  |  |  |  | |
|  | Caudal anterior cingulate | 2.27±0.15 | 2.37±0.15 | 0.004 | 0.67 | |
|  | Caudal middle frontal | 2.36±0.11 | 2.47±0.11 | < 0.001 | 1.00 | |
|  | Cuneus | 1.83±0.13 | 1.89±0.10 | 0.041 | 0.52 | |
|  | Entorhinal | 2.90±0.20 | 3.05±0.26 | 0.004 | 0.66 | |
|  | Fusiform | 2.52±0.12 | 2.63±0.10 | < 0.001 | 0.95 | |
|  | Inferior parietal | 2.27±0.10 | 2.36±0.10 | < 0.001 | 0.91 | |
|  | Inferior temporal | 2.57±0.12 | 2.65±0.08 | 0.002 | 0.77 | |
|  | Isthmus cingulate | 2.10±0.14 | 2.23±0.13 | < 0.001 | 0.94 | |
|  | Lateral occipital | 2.04±0.10 | 2.13±0.09 | < 0.001 | 0.87 | |
|  | Lateral orbitofrontal | 2.43±0.11 | 2.55±0.08 | < 0.001 | 1.17 | |
|  | Lingual | 1.92±0.12 | 1.99±0.10 | 0.019 | 0.61 | |
|  | Medial orbitofrontal | 2.24±0.10 | 2.35±0.09 | < 0.001 | 1.11 | |
|  | Middle temporal | 2.66±0.12 | 2.74±0.11 | 0.001 | 0.75 | |
|  | Parahippocampal | 2.56±0.20 | 2.68±0.19 | 0.039 | 0.59 | |
|  | Paracentral | 2.29±0.15 | 2.42±0.12 | < 0.001 | 0.88 | |
|  | Parsopercularis | 2.46±0.15 | 2.57±0.09 | < 0.001 | 0.84 | |
|  | Parsorbitalis | 2.56±0.16 | 2.65±0.12 | 0.020 | 0.59 | |
|  | Parstriangularis | 2.36±0.12 | 2.46±0.11 | < 0.001 | 0.91 | |
|  | Pericalcarine | 1.62±0.14 | 1.66±0.13 | 0.255 | / | |
|  | Post-central | 2.01±0.11 | 2.09±0.09 | 0.002 | 0.75 | |
|  | Posterior cingulate | 2.24±0.13 | 2.37±0.11 | < 0.001 | 1.11 | |
|  | Precentral | 2.44±0.14 | 2.56±0.11 | < 0.001 | 0.89 | |
|  | Precuneus | 2.24±0.12 | 2.34±0.10 | < 0.001 | 0.92 | |
|  | Rostral anterior cingulate | 2.57±0.13 | 2.64±0.10 | 0.025 | 0.57 | |
|  | Rostral middle frontal | 2.22±0.11 | 2.33±0.08 | < 0.001 | 1.01 | |
|  | Superior frontal | 2.51±0.11 | 2.64±0.10 | < 0.001 | 1.16 | |
|  | Superior parietal | 2.08±0.10 | 2.18±0.10 | < 0.001 | 1.03 | |
|  | Superior temporal | 2.65±0.15 | 2.79±0.10 | < 0.001 | 1.03 | |
|  | Supramarginal | 2.39±0.12 | 2.47±0.09 | 0.003 | 0.71 | |
|  | Frontal pole | 2.55±0.17 | 2.67±0.17 | 0.004 | 0.70 | |
|  | Temporal pole | 3.42±0.26 | 3.52±0.26 | 0.265 | / | |
|  | Insula | 2.78±0.13 | 2.84±0.10 | 0.017 | 0.52 | |
| ***Regional volume*** (mm3) | |  |  |  |  | |
|  | Cerebellum White Matter (x 10^3^) | 13.83±2.39 | 13.96±1.89 | 0.427 | / | |
|  | Cerebellum Cortex (x 10^3^) | 49.58±6.03 | 48.72±5.08 | 0.895 | / | |
|  | Thalamus (x 10^3^) | 6.76±1.19 | 6.79±1.05 | 0.981 | / | |
|  | Caudate (x 10^3^) | 3.52±0.52 | 3.43±0.56 | 0.488 | / | |
|  | Putamen (x 10^3^) | 4.78±0.84 | 4.78±0.71 | 0.966 | / | |
|  | Pallidum (x 10^3^) | 1.77±0.29 | 1.76±0.22 | 0.787 | / | |
|  | Hippocampus (x 10^3^) | 4.04±0.50 | 4.03±0.59 | 0.789 | / | |
|  | Amygdala (x 10^3^) | 1.66±0.24 | 1.71±0.27 | 0.302 | / | |
|  | Data are reported as mean ± Standard deviation  Comparison in-between groups have been performed GLM with sex, and age as covariates.  The effect size is reported as Cohen’s *d* obtained as the difference of the means divided by the standard deviation of the data.  NBV = normalized brain volume; NGMV = normalized grey matter volume; DGMV = deep-grey matter volume. | | | | |  |
